# Stretching Morphogenesis of the Roof Plate and Formation of the Central Canal

**DOI:** 10.1371/journal.pone.0056219

**Published:** 2013-02-07

**Authors:** Igor Kondrychyn, Cathleen Teh, Melvin Sin, Vladimir Korzh

**Affiliations:** 1 Institute of Molecular and Cell Biology, A-STAR, Singapore, Singapore; 2 Department of Biological Sciences, National University of Singapore, Singapore, Singapore; Instituto Gulbenkian de Ciência, Portugal

## Abstract

**Background:**

Neurulation is driven by apical constriction of actomyosin cytoskeleton resulting in conversion of the primitive lumen into the central canal in a mechanism driven by F-actin constriction, cell overcrowding and buildup of axonal tracts. The roof plate of the neural tube acts as the dorsal morphogenetic center and boundary preventing midline crossing by neural cells and axons.

**Methodology/Principal Findings:**

The roof plate zebrafish transgenics expressing cytosolic GFP were used to study and describe development of this structure *in vivo* for a first time ever. The conversion of the primitive lumen into the central canal causes significant morphogenetic changes of neuroepithelial cells in the dorsal neural tube. We demonstrated that the roof plate cells stretch along the D–V axis in parallel with conversion of the primitive lumen into central canal and its ventral displacement. Importantly, the stretching of the roof plate is well-coordinated along the whole spinal cord and the roof plate cells extend 3× in length to cover 2/3 of the neural tube diameter. This process involves the visco-elastic extension of the roof place cytoskeleton and depends on activity of Zic6 and the Rho-associated kinase (Rock). In contrast, stretching of the floor plate is much less extensive.

**Conclusions/Significance:**

The extension of the roof plate requires its attachment to the apical complex of proteins at the surface of the central canal, which depends on activity of Zic6 and Rock. The D–V extension of the roof plate may change a range and distribution of morphogens it produces. The resistance of the roof plate cytoskeleton attenuates ventral displacement of the central canal in illustration of the novel mechanical role of the roof plate during development of the body axis.

## Introduction

It is thought that neurulation ends after the neural tube is formed [1], [2]. Once formed the neural tube could be divided from dorsal to ventral into the roof plate (RP), alar plate, basal plate and floor plate. The RP is an embryonic organizing center that occupies the dorsal midline of the vertebrate neural tube along the entire anterior-posterior (A–P) axis, where it produces morphogens responsible for dorsal cell fates, including BMP and Wnt [3]–[7]. In addition, RP also acts as a barrier preventing axons and cells migrating across the dorsal midline [8], [9].

RP cells share origin with the neural crest (NC) cells, dorsal interneurons, choroid plexus and meninges [3], [10]–[12]. While it was shown that the RP elongates during conversion of the primitive lumen into the central canal [9], [13], there are no detailed studies describing this complex process *in vivo*. Historically, a relationship between the stretching of the RP and conversion of the primitive lumen into the central canal was a matter of some controversy. For example, Böhme [14] concluded that during cat development the formation of the central canal does not result directly in the formation of the dorsal glial septum (a.k.a. RP). In contrast, Sevc et al. [13] recognized interdependence of RP elongation and conversion of the primitive lumen into the central canal in rat, but suggested that the rearrangement and migration of radial glial cells is behind the transformation of the primitive lumen into the central canal. These authors along with others also recognized a role of two other factors behind conversion of the primitive lumen into central canal: cell proliferation in the neural tube and build-up of axonal pathways. Importantly, the cells lining the ventricular surface display unique organization of actin microfilaments, which disturbance results in abnormal neurulation [1], [15]–[18]. Hence an idea of apical constriction of neuroepithelial cells of the neural plate driven by contraction of an apical meshwork of filamentous actin (F-actin) involving the vimentin-positive intermediate filaments-based cytoskeleton [19] as a main driving force of neurulation is well developed and validated experimentally [20]–[22]. Further to that a number of mutations affecting genes regulating organization of actin cytoskeleton cause neural tube defects and some of these mutants are characterized by the abnormal body axis. Therefore, the genes affecting actin function are under study as candidates for anencephaly-risk genes in humans [23]–[28]. The lack of obvious *spina bifida* phenotype in mouse mutants affecting neurulation anteriorly led to suggest that normal actin function is critical for cranial rather than caudal neural tube closure in mice [29].

The Zic family of zinc-finger proteins is known for its crucial role in neural development and disease and, in particular, in control of neurulation (reviewed in [30]–[32]). Dandy-Walker malformation caused by heterozygous loss of Zic1 and Zic4 in human is defined by deficiency of the dorsal neural tube, including hypoplasia and upward rotation of the cerebellar vermis and cystic dilation of the fourth ventricle. This condition is phenocopied by similar genetic anomaly in mice [33]–[35]. Since it was shown that in zebrafish Zic1 and Zic4 control expression of the roof plate determinant Lmx1b, the defects in human patients deficient in these genes could be due to abnormal development of the roof plate [36]. Importantly, two other proteins of the same family, Zic2 and Zic5 are involved in neurulation during formation of the dorso-lateral hinge points, where they are required for apical F-actin and active myosin II localization and junction integrity [37]. Being lost in terrestrial vertebrates, Zic6 is probably the most mysterious member of the Zic family [38]–[40].

Our analysis of roof plate morphogenesis during conversion of the primitive lumen into the central canal in developing zebrafish for a first time illustrated this process in vertebrates *in vivo.* It revealed a novel mechanical role of the roof plate cytoskeleton, which attenuates the forces driving formation of the central canal. Here Zic6 plays a role in regulation of RP cytoskeleton and, in particular, attachment of these cells to the apical complex of proteins at the surface of the central canal.

## Results

### SqET33 Transgenic Line Expresses GFP in RP Cells

**Figure 1:**
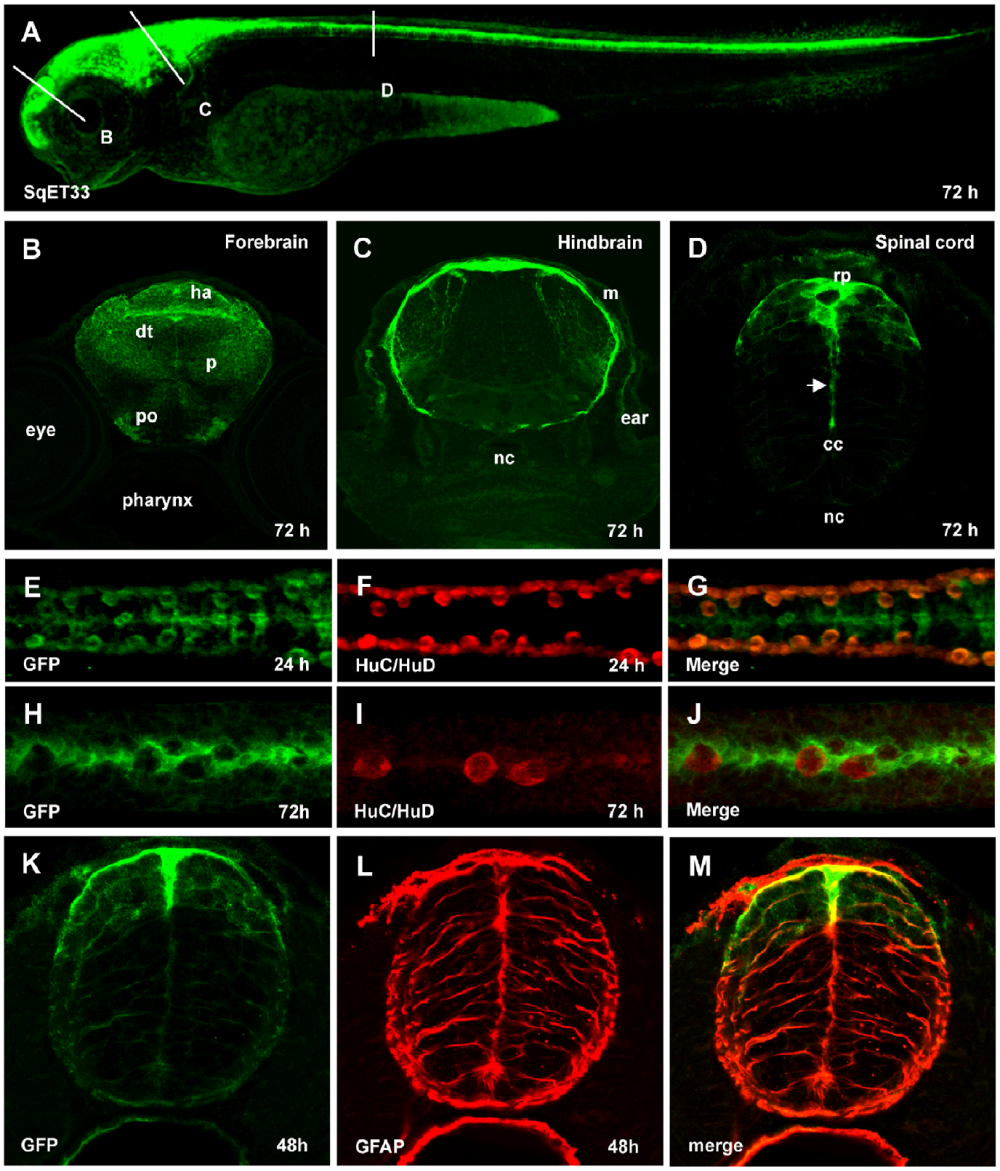
Characterization of SqET33 transgenic line. (A), Confocal image of 3 dpf larva of SqET33 line, lateral view. Dashed lines depict the position of transverse sections shown in B–D. (B–D), transverse sections, immunofluorescent staining with anti-GFP antibodies. Arrow indicates the elongated central process of the roof plate cell. (E–J), whole-mount immunofluorescent staining with anti-GFP (midline RP cells and lateral dorsal interneurons) and anti-HuC/HuD (lateral dorsal neurons) antibodies, dorsal view of spinal cord. (K–M), immunofluorescent staining with anti-GFP (green) and anti-GFAP (red) antibodies, transverse section of the spinal cord. Abbreviations: cc, central canal; dt, dorsal thalamus; ha, habenula; m, meninx; nc, notochord; p, pallium; po, preoptic area; rp, roof plate.

The SqET33 transgenic line used in this study has been established during *Tol2* transposon-mediated enhancer trap screen [38]. In the 3 days-old larva GFP fluorescence is detected in the neural tube along the A–P axis (Fig. 1A) largely in the dorsal aspect of the forebrain (Fig. 1B), midbrain, hindbrain (Fig. 1C) and spinal cord (Fig. 1D). In the brain, the laterally elongating processes of GFP-positive cells spread around the neural tube forming its outer envelope, the meninx (Fig. 1C). In the spinal cord, the dorsal midline GFP-positive cells elongate along the midline in ventral direction, while maintaining a contact with the primitive lumen throughout its conversion into the central canal. During this process they develop a palisade of extensions (Fig. 1D and see below). The dorsal midline GFP-positive cells are non-neuronal, since they do not express neuronal marker HuC/HuD (Fig. 1E–J). Their phenotype is reminiscent of radial glia and they express GFAP (glial fibrillar acidic protein) (Fig. 1K–M), a known marker for RP cells and astroglia in the rodent spinal cord [9]. In the zebrafish neural tube radial glia cells are mitotically active and also express GFAP [41], [42]. Based on their midline position, morphology, GFAP expression and lack of neuronal markers, we concluded that they represent the RP cells.

**Figure 2:**
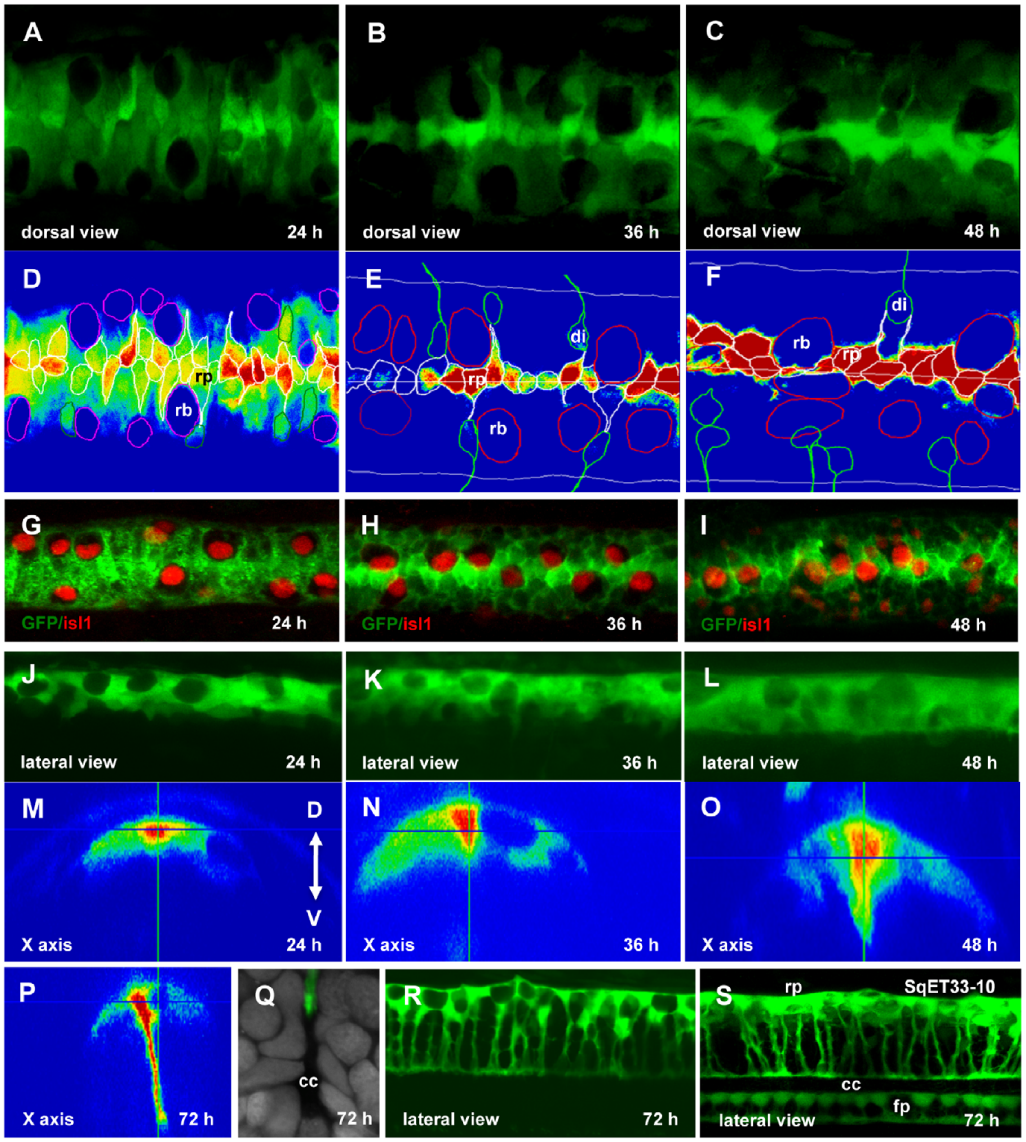
Re-orientation of the RP cells. Confocal images of the spinal cord of SqET33 line at different developmental stages (A–C, dorsal view, J–L, R, lateral view, M–P, orthogonal optical sections) and outline of the dorsal cells (D–F) superimposed onto the GFP intensity chart. Note that RB cells undergo the lateral-medial displacement. (G–I), Whole-mount immunohistochemistry detecting Islet1 in RB cells (red, nuclei), dorsal view of the spinal cord. (Q), Transverse section of the spinal cord at high magnification showing a fine structure of the central canal. RP process is stained with anti-GFP (green) and nuclei are counterstained with DAPI (grey). (S), Confocal image of the spinal cord of SqET33-10 line expressing GFP in the roof and floor plates, lateral view. Abbreviations: cc, central canal; di, dorsal interneurons; fp, floor plate; rb, Rohon-Beard cells; rp, roof plate cells.

During development the RP undergoes major morphogenetic rearrangement. The Isl1-positive sensory Rohon-Beard (RB) cells shift dorso-medially squeezing between the RP cells (Fig. 2A–I). Somata of RP cells, which were oriented medio-laterally at 24 hpf (Fig. 2A, D and J, M), by 36 hpf re-orient along the dorso-ventral (D–V) axis (Fig. 2K, N). At around 48–51 hpf RP cells start elongating (Fig. 2L, O) and by 65–66 hpf form long ventrally directed extensions (Fig. 2P–R and Movie S1). This process could be described as the uniform stretching well-coordinated along the whole A–P axis of the spinal cord. As the stretching slows down, the early (fast) phase of extension is replaced by the slow phase correlating with increase of the neural tube diameter due to growth. The length of the RP triples from 17.6 to 51.5 µm (Fig. S1). The endfeet of the central processes now form the dorsal surface of the central canal (Fig. 2Q), which is even more obvious in the SqET33-10 transgenics [38], [40] expressing GFP in both roof and floor plates (Fig. 2S). It is only upon its extension that the RP may perform it barrier function and prevent cells and axons from crossing the midline (Movie S2). Unlike the RP, the floor plate cells extend much less. The palisade formed by the RP processes was observed as late as 12 dpf (data not shown), the latest time point inspected.

### The Stretching of RP Correlates with Recession of the Primitive Lumen and Formation of the Central Canal

Given a connection of the RP cells to the central canal, we asked whether the RP stretching correlates with conversion of the primitive lumen of the spinal cord into the central canal. This process also known as “obliteration” of the primitive lumen was described in cat, mouse and rat [14], [43], [44]. It is accompanied by formation of the dorsal glial septum represented by the RP and other cells [9], [13]. In zebrafish prior to 24 hpf two continuous apical membranes form along the midline and define the inconspicuous primitive lumen or neurocoel extending along all spinal cord [45]. The apical membranes present a useful landmark to study the elongation of the RP.

**Figure 3:**
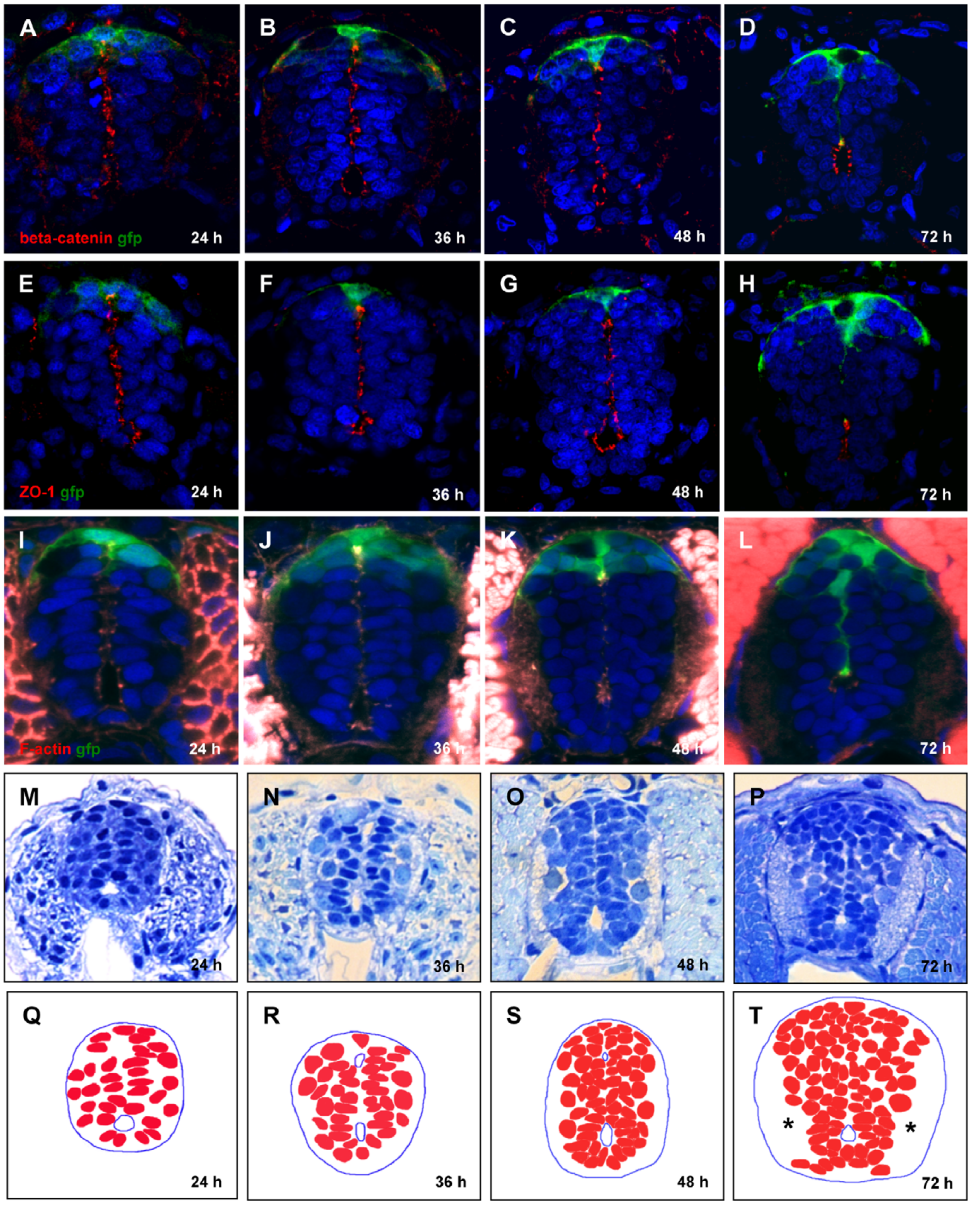
Conversion of primitive lumen into central canal. (A–L), Contraction of opposed apical surfaces reflects recession of the primitive lumen into a central canal between 24 and 72 hpf. Immunofluorescent staining by a combination of anti-GFP with anti-β-catenin antibody (A–D), anti-ZO-1 antibody (E–H), and phalloidin that detects F-actin (I–L). Increase in cell number and build-up of axonal tracts (*), plastic transverse sections of the spinal cord (M–P). (Q–T), Schematics corresponding to (M–P) showing cell nuclei.

We asked, what is a relationship between lumen recession and extension of the RP? As lumen receded the expression of proteins representing the apical complex at the surface of the central canal - β-catenin, ZO-1 and F-actin has changed: the long slit-like domain became small, round and shifted ventrally. The processes of RP cells attached to the lumen stretched along with its closure (Fig. 3A–L). Thus, the RP stretching morphogenesis correlates with conversion of the primitive lumen into the central canal. What are the factors behind stretching of the RP? It was suggested that obliteration of primitive lumen takes place due to an increase in a number of neural cells in the spinal cord and an expansion of territory occupied by white matter [14]. Indeed, during this period a number of cells in the spinal cord increased as manifested by an increasingly dense packing of nuclei resulting from an increase in a number of cells, and the buildup of the acellular white matter in the lateral aspect of the neural tube (Fig. 3M–T). In result, the whole cellular territory acquired the wedge shape extended along the D–V axis. Thus, F-actin constriction, cell proliferation and buildup of white matter correlate with the rearrangement of the primitive lumen into central canal and stretching of the RP cells. These could be the mechanistic factors behind late neurulation in zebrafish similar to that in mammals.

### Genetic Analysis of RP Elongation

**Figure 4:**
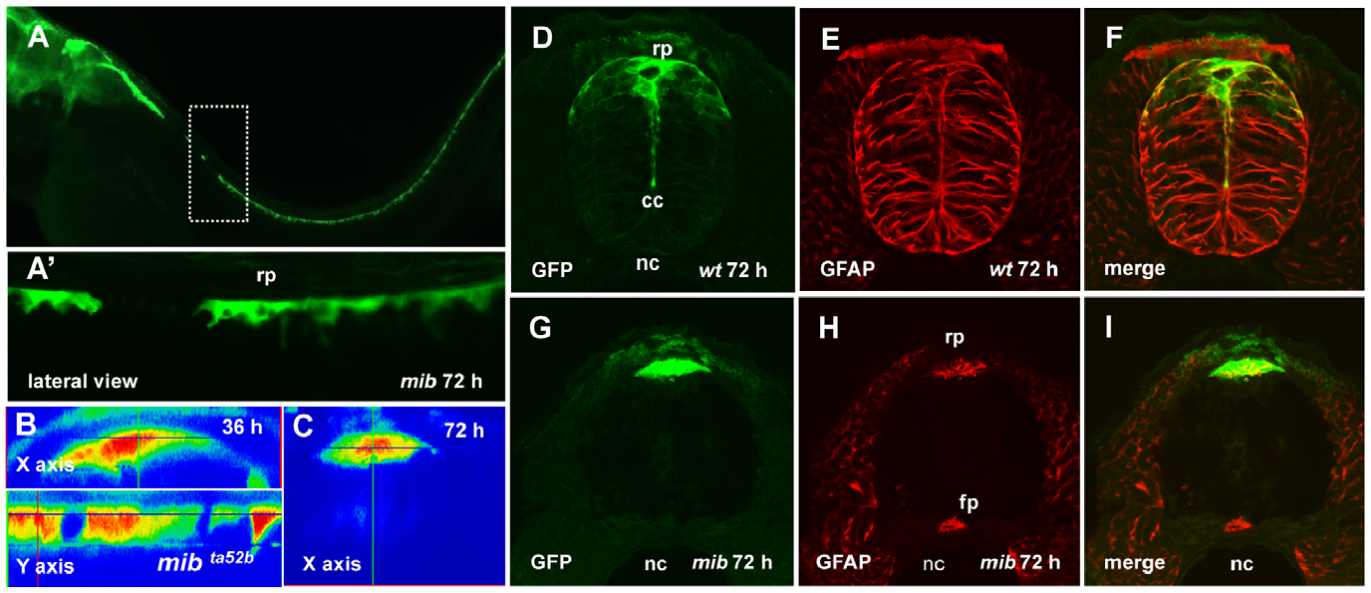
The roof plate formation in the *mib* mutant. (A), Confocal images, lateral view of the spinal cord of *mib* mutant, 72 hpf. Dashed rectangular shows the magnified in (A′) region of the spinal cord with the absence of GFP-positive RP cells. (B, C), the orthogonal optical sections of confocal images of the spinal cord of *mib* mutant illustrate lack of the roof plate extension between 36 and 72 hpf. Immunofluorescent staining of the transverse sections of spinal cord using anti-GFP (green) and anti-GFAP (red) antibodies; wild-type embryo (D–F) and *mib* mutant (G–I).

It was shown that mutations affecting components of the Nodal and Hedgehog (Hh) signaling pathways disturb formation of the neurocoel and floor plate [46]–[49]. We asked whether these and some other pathways contribute in RP elongation. Hence, we analyzed this process in the spinal cord of mutants affecting Hh (*smu* and *syu*), Nodal (*oep*), Notch (*mib*) and Par (*pard6γb*, see below) functions at the background of SqET33 transgenics. Mutations affecting the Hh and Nodal signaling caused deformation of the body axis in *smu*, *syu* and *oep* mutants (curved down trunk). However, the GFAP-positive radial scaffold is formed and the RP stretched, although the overall length of RP processes was shorter in comparison with controls (Fig. S1). In contrast, *mib* mutant showed the curved up body axis (Fig. 4A), loss of anterior RP cells (Fig. 4A′) as well as an absence of RP stretching (Fig. 4B, C) and GFAP-positive radial scaffold (Fig. 4D–I). Thus, the reduction of the primitive lumen and extension of the RP in zebrafish correlates with formation of the GFAP-positive radial scaffold similar to that in mammals [13]. In contrast, the direction of the body curvature in different mutants shows no correlation with the RP extension.

**Figure 5:**
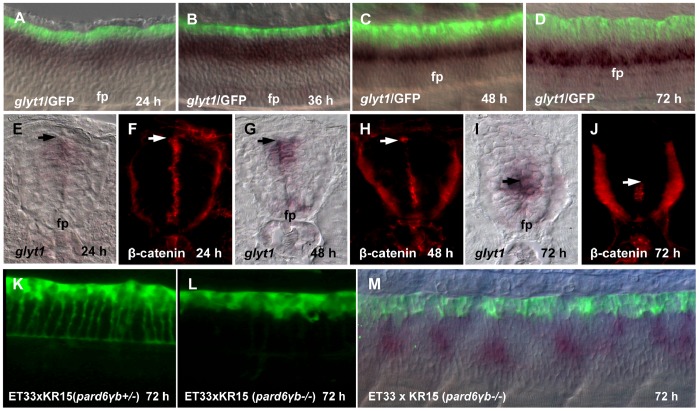
Growth of the RP processes correlates with a shift of *glyt1* expression along D–V axis. (A–D), Double whole mount *in situ* hybridization (*glyt1*, dark purple) and immunostaining (GFP, green). *glyt1* is expressed in the midline glial cells except RP; GFP is expressed in the RP cells. (E–J), Whole mount *in situ* hybridization (*glyt1*, dark purple) and immunostaining (β-catenin, red), the transverse sections of the spinal cord. The arrow shows an approximate position of attachment of the RP process to the apical surface of central canal. Confocal images of the spinal cord of *pard6γb* heterozygote (K) and *pard6γb* mutant (L) transgenic fish. (M), double whole mount *in situ* hybridization (*glyt1*, dark purple) and immunostaining (GFP, green) of *pard6γb* mutant. Abbreviation: fp, floor plate.

Glyt1 (Slc6a9) is a glycine transporter expressed in non-neuronal cells that face the lumen of the developing spinal cord [50]. The ventricular zone contains radial glial cells that function as neural stem cells [51]–[52]. Glycine is a crucial element in rapid proliferation of cancer cells [53]–[55]. During development the expression domain of *glyt1* shifts ventrally in parallel with conversion of the primitive lumen into the central canal as illustrated by RP extension (Fig. 5A–D) and dynamic expression of β-catenin (Fig. 5E–J). At 72 hpf *glyt1* is expressed in cells lateral to the central canal, i.e. immediately adjacent to the ventral portion of RP extensions (Fig. 5I, J).

The mutation in *pard6γb* gene disturbs formation of the central canal resulting in multiple lumens [56]. We developed recently another mutant allele of this gene - transgenics SqKR15 line that carry the *Tol2* transposon insertion in *pard6γb* gene [57]. As expected in SqKR15 homozygote embryos *glyt1* was expressed as multiple short domains in illustration of abnormal central canal presented as multiple small lumens. In parallel, the RP failed to stretch (Fig. 5K–M). This illustrated the fact that abnormal conversion of the primitive lumen into central canal affects stretching morphogenesis of the RP.

**Figure 6:**
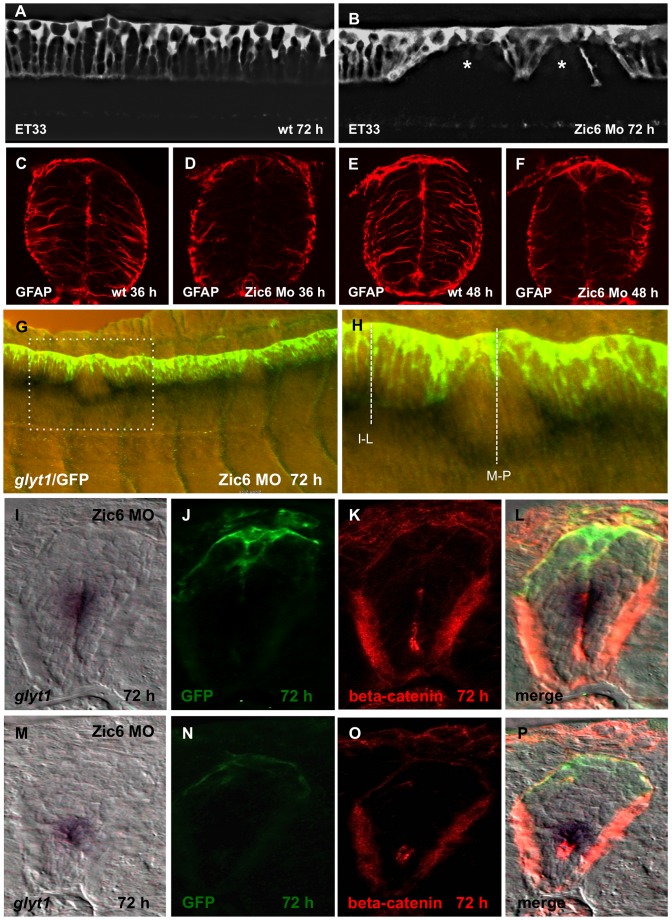
Zic6 is required for development of the radial glial scaffold and attachment of RP cells to the dorsal surface of central canal. Confocal images, lateral view of the spinal cord, 72 hpf. SqET33, control (A) and Zic6 morphant (B). The asterisk indicates the “gaps” in the palisade of RP cells. (C–F), Immunostaining of the transverse sections of the spinal cord with anti-GFAP antibody. (G), Double whole-mount *in situ* hybridization (*glyt1*, dark purple) and immunostaining (GFP, yellow/green) of the spinal cord Zic6 morphant. The dashed rectangular marks the magnified region (H). The dashed lines show the approximate positions for the transverse sections shown in (I–P). (I–P), distribution of *glyt1*, β-catenin and GFP in two positions in the spinal cord of Zic6 morphants shown in H, corresponding to I–L and M–P.

SqET33 carries a single transposon insertion in *zic6* 3′UTR. In this line GFP expression recapitulates that of *zic6* [38] expressed in the RP (Fig. S2). We asked whether this gene plays a role in RP development. In absence of the Zic6 mutant we used the morpholino (MO)-mediated loss-of-function (LOF) approach. Zic6 morphants developed the curled-down body axis (Fig. S3) and some of them consistently showed the characteristic “gaps” in the palisade of the RP extensions at the level of extended yolk, where extensions of the RP cells were short and their distribution abnormal (Fig. 6A, B). In parallel, the GFAP-positive radial processes failed to form similar to that in *mib* mutants (Fig. 6C–F).

Some understanding of the mechanism involved in formation of “gaps” in the roof plate of Zic6 morphants came in result of analysis of distribution of *glyt1* mRNA and β-catenin. Intuitively, we expected to find the expression domains of GFP and *glyt1* in close proximity to each other all along the A–P axis, including the “gaps”. Contrary to these expectations at the “gap” level the two markers separated with domain of *glyt1* expression shifted ventrally and short processes of RP cells found in abnormally dorsal position (Fig. 6G–P). in parallel, GFP expression in the roof plate was no longer found to be associated with β-catenin signal with the latter being displaced ventrally (Fig. 6K, L and O, P). This phenotype is consistent with a breakdown of the attachment of RP processes to the central canal and their displacement dorsally due to contraction in parallel with displacement of the *glyt1* and β-catenin domains of expression ventrally.

**Figure 7:**
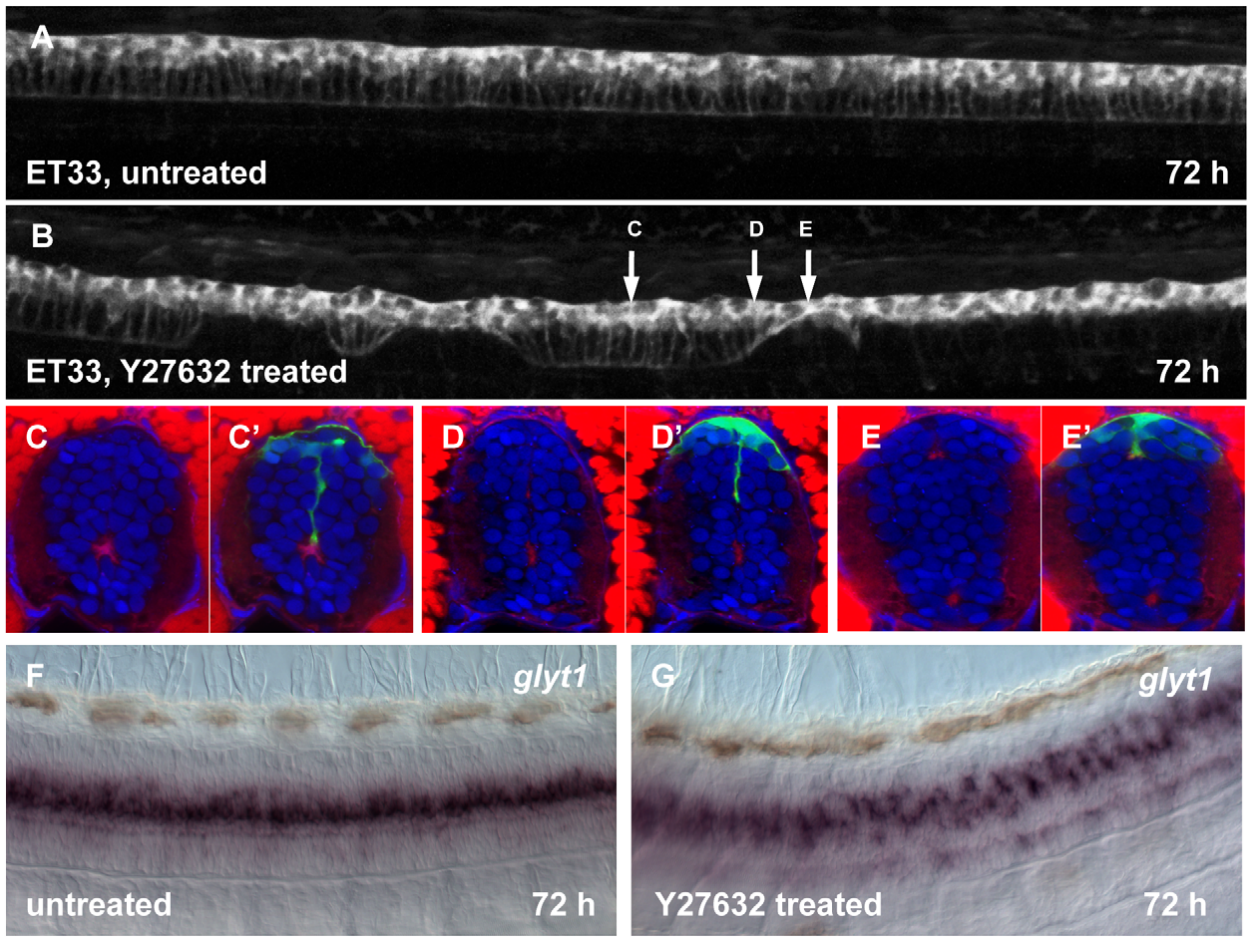
Effect of Rock inhibition on the RP morphogenesis. Confocal images of 72 hpf larva, untreated (A) and after Y-27632 injection into hindbrain ventricle at 30 hpf (B). The arrows show an approximate position of the transverse sections. (C–E′), transverse sections of the spinal cord of larva, treated with Y-27632. Immunofluorescent staining with anti-GFP (green) and phalloidin (red). Whole mount *in situ* hybridization with *glyt1* antisense RNA probe, control (F) and after treatment with Y-27632 (G).

Several features of the morphant phenotype - the curled body axis, failure of RP extension and GFAP expression as well as changes in *glyt1*/β-catenin expression were consistent with defects of the cytoskeleton in RP cells. Hence we performed a small-scale screen for inhibitors of various signaling pathways in attempt to mimic the effect of Zic6 LOF. The inhibitors were injected into the hindbrain ventricle at 30 hpf from where they spread throughout the whole ventricular system and central canal [58]. The defects in RP morphology were scored at 72 hpf. Only embryos injected with the inhibitor of Rho-associated protein kinases (Rock1 and Rock2) - Y27632 [59]–[61] showed somewhat similar phenotype (Fig. 7A, B). Similar to that in Zic6 morphants, an inhibition of Rock caused a failure of RP extension in parallel with changes in distribution of proteins of the apical protein complex (F-actin, Fig. 7 C–E) and distribution of *glyt1* expression (Fig. 7F, G). The difference was manifested by much larger gaps in the roof plate palisade or complete failure of RP extension.

Rock activation is known to result in a concerted series of events at the level of cytoskeleton that promote force generation and morphological changes in particular during neurulation [60]. An application of inhibitors blocking individual contractile proteins (F-actin, myosin-2, tubulin) that were shown earlier to affect neural tube closure [60] failed affect extension of the RP (Table S1). This could be due to insufficient penetration of these compounds into the lumen of the neural tube resulting in their suboptimal concentration or due to redundancy at the level of individual contractile proteins involved in this process.

**Figure 8:**
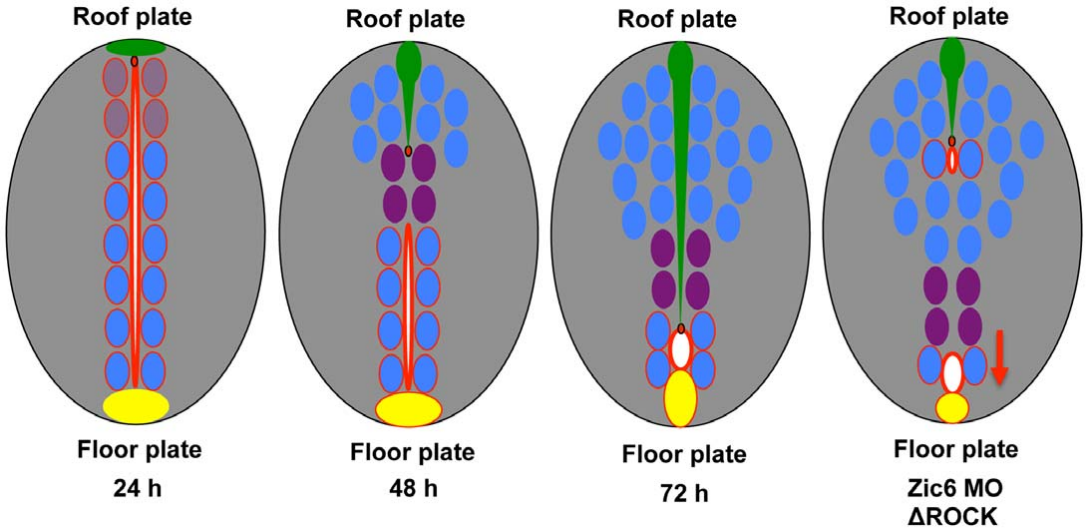
Critical events during stretching morphogenesis of the RP in zebrafish. The scheme illustrates the stretching morphogenesis of the RP, which takes place during late neurulation in correlation with reduction of the primitive lumen into the central canal. In zebrafish this process depends on activity of Zic6 and Rock.

Here we demonstrated that extension of the RP cells requires their attachment to the apical complex of proteins at the surface of the central canal and depends on activity of Zic6 and Rho-associated protein kinase. The resistance of the RP cytoskeleton attenuates ventral displacement of the central canal during a process of reduction of the primitive lumen (Fig. 8).

## Discussion

The RP is an evolutionarily conserved dorsal organizing center of the embryonic neural tube and the midline barrier [3], [8]–[10]. The RP development was studied in birds and mammals, but details of morphogenesis of these cells are not fully understood in particular in a context of conversion of the primitive lumen into the central canal. Here we used several zebrafish transgenics with expression of cytosolic GFP in the RP to study for the first time the morphogenesis of the RP cells *in vivo* and analyze the factors involved. The deficiency of the roof plate cells adhesion results in interruption of conversion of the primitive lumen into the central canal.

The RP undergoes well-coordinated “stretching” morphogenesis along the whole extent of the spinal cord (Movie S1) in correlation with closure of the primitive lumen. This probably is the first morphogenetic event coordinated within the whole spinal cord. Noteworthy that initiation of fast RP extension correlates with hatching (48–52 hpf) [62]. Our observations were made on larvae that were dechorionated prior to imaging. Hence the reduction of the primitive lumen and stretching of the RP are independent of the hatching *per se*. These developmental events must be regulated by distinct genetic mechanisms.

The stretching morphogenesis of the RP takes place under the influence of opposing forces. Here a constriction of the apical belt of F-actin along with cell overcrowding and lateral pressure of expanding axonal pathways (i.e. pull-push force) drives an elongation of the cellular (grey) area of the neural tube along the D–V axis. This correlates with conversion of the primitive lumen into a central canal. The resistance (i.e. dragging force) of cytoskeleton of the RP and other cells of the neural tube undergoing visco-elastic stretching attenuates this process. In zebrafish this process is rather asymmetrical with much more significant elongation of the roof plate comparing to the floor plate. This is similar to that in cat, where cell proliferation along with a buildup of axonal tracts significantly contributes into dorsal closure (“obliteration”) of primitive lumen with little extension of the floor plate [14]. Similar developmental scenario takes place in the spinal cord in camel [63]. This is somewhat different from that in mice and rat, where the closure of primitive lumen happens both dorsally and ventrally [13], [41]. In this respect, this process in zebrafish is more reminiscent of that in the cat and camel.

Based on analysis of primitive lumen closure in mammals, Sevc et al. [13] proposed an active role of radial glia in the transformation of the primitive lumen into the central canal. The RP morphogenesis should be considered in view of interaction of two opposing forces, where the cytoskeleton in cells that elongate counteracts the pull-push forces driving conversion of the primitive lumen into the central canal. Hence mechanically a role of RP cells manifests itself through cytoskeleton resistance opposing the factors behind the push-pull force. To form an integrated layer the cells lining the ventricular surface rely on their cytoskeleton and the apical complex of proteins, including F-actin, ZO-1 and β-catenin [1], [15]–[18]. Zic6 fits this model nicely. Other proteins of the Zic family either control formation of the roof plate (Zic1 and Zic4) or are involved in neurulation during formation of the dorso-lateral hinge points (Zic2a and Zic5), where they are required for junction integrity [36], [37]. Perhaps, during closure of the primitive lumen and formation of the central canal Zic6 plays a role in regulating the cytoskeleton of RP. The redundancy of Zic proteins may explain why a “gap” phenotype was detected only in some embryos. This explanation is also in line with conservation of the neurulation mechanism involving closure of the primitive lumen and formation of the central canal during evolution despite disappearance of Zic6 in tetrapods [13], [32], [37]–[39], [64].

Our data support a model suggesting that during conversion of the primitive lumen into central canal the resistance of RP cytoskeleton, and by extension that of other cells in the dorsal neural tube, which are connected by the apical complex of proteins at the surface of the central canal, is insufficient to resist the factors behind the pull-push force causing stretching of the RP (Fig. 8). The failure of adhesion of the RP cells to the apical complex of proteins at the surface of the central canal in Zic6 morphants causes a breakdown between cytoskeleton and apical junction resulting in displacement of the roof plate dorsally and the central canal ventrally. It also weakens the cytoskeleton of the spinal cord resulting in curling of the body axis. This elegantly illustrates a tug-of-war between different axial structures behind straightening of the body axis and demonstrates the novel mechanical function of the roof plate during formation of the body axis.

## Materials and Methods

Fish were maintained, mated and raised as described [65] according to the rules of the IMCB fish facility and the Biopolis IACUC protocol #090430. Embryos were kept at 28.5°C and staged according to Kimmel et al. [62]. For anesthetizing, 0.16 mg/ml of buffered 3-aminobenzoic acid ethyl ester (Tricaine, Sigma), containing Tris buffer, pH7, was used. To inhibit pigment formation, 0.2 mM 1-phenyl-2-thiourea was added to embryo medium after 16 hours of post fertilization (hpf).

### Transgenic and Mutant Fish Lines

In this study we used the following transgenic lines: SqET33, SqET33-10 and Sq33-E20, generated during enhancer trap screen [11], [38], [40]. Several lines with different mutant background were generated by mating the SqET33 line with heterozygous mutant partners. The offspring were raised and heterozygous transgenic lines carrying the appropriate mutated allele were identified by mating with given mutant. The following alleles of zebrafish mutants were used in this study: *mib^ta52b^*, *oep^z1^*, *smu^b641^*, *syu^t4^* and SqKR15 harboring transposon insertion in *pard6γb*, which represents an independent mutant allele affecting this gene [57], [66]–[69].

### Cloning of *glyt1* Gene

Full-length cDNA for *glyt1* gene was amplified using primers: *forward* 5′-AGA CTG ACC TGG AGA GTG TTT TGC C-3′ and *reverse* 5′-CAA TGC GCT ATA TAC ACA ATG CGT G-3′. Resulting PCR product was cloned into pGEM-TEasy vector (Promega).

### Antibodies

The following primary antibodies were used: mouse monoclonal anti-GFP (clone B-2, Santa Cruz Biotechnology), anti-β-catenin (clone 15B8, Sigma), anti-GFAP (clone G-A-5, Sigma) and anti-ZO1 (gift from Dr. Drummond, Harvard University, USA); rabbit polyclonal anti-Isl-1 [70] and anti-GFP (BD Living Colors, Clontech). Secondary antibodies from Molecular Probes were used: Alexa Fluor 488-conjugated goat anti-mouse and anti-rabbit F(ab)_2_ fragments and Alexa Fluor 543-conjugated goat anti-mouse and anti-rabbit F(ab)_2_ fragments.

### Whole-mount *in situ* Hybridization

Embryos were fixed in Histochoice Tissue Fixative MB (Amresco, USA) overnight at room temperature, washed in PBST and treated with 3% H_2_O_2_ for 15 min. Hybridization was carried out in hybridization solution containing 5% dextran sulphate at 65°C. After stringency wash, the digoxigenin-labelled RNA probe was detected with anti-digoxigenin antibody, conjugated with alkaline phosphatase (Roche). Staining was developed in detection buffer containing 2% polyvinyl alcohol (Sigma).

### Whole-mount Immunofluorescent Histochemistry

Embryos were fixed in Histochoice Tissue Fixative MB (Amresco, USA) for 2 hours at room temperature, washed 3 times for 15 min in PBST (1 x PBS, 0.1% Tween-20) and permeabilized in 0.1% Triton X-100 in 0.1% sodium citrate for 30 min. Then embryos were incubated for 2 hours in 5% Blocking Reagent (Roche) in MAB (150 mM maleic acid, pH 7.5, 100 mM NaCl, 0.1% Tween-20). Embryos were incubated with primary antibodies (1∶200) overnight at 4°C, washed 4 times for 30 min in MAB and incubated with secondary antibodies (1∶500) for 6 hours at room temperature in darkness. Finally, embryos were extensively washed in PBS and kept in 50% glycerol in PBS until being monitored under fluorescent or confocal microscope.

### Immunofluorescent Histochemistry

Embryos were fixed in Histochoice Tissue Fixative MB (Amresco, USA) overnight at 4°C, washed 3 times for 15 min in PBST and embedded in 2% agar (Difco, USA) in PBS. For F-actin staining, embryos were fixed with 4% paraformaldehyde in actin stabilizing buffer (50 mM PIPES, 150 mM KCl, 1 mM CaCl_2_, 1 mM EGTA, pH 6.5) overnight at 4°C. Before cryosectioning, agar blocks were kept in 30% sucrose in PBS overnight at 4°C. Cryosections with thickness 15µm were done using Leica cryostat. Sections were washed 3 times for 5 min in PBST, permeabilized for 30 min in PBS containing 0.1% Triton X-100 and1% DMSO, blocked in blocking solution (5% BSA and 0.05% Tween-20 in PBS) for 1 hour and incubated with primary antibodies (1∶200) for 1 hour in humid chamber at room temperature. Then sections were rinsed 3 times for 5 min in PBST and incubated with secondary antibodies (1∶500) for 1 hour. F-actin was detected using phalloidin-Alexa Fluor 635 (Molecular Probes) according to manufacture’s protocol. Finally, sections were washed 3 times for 5 min in PBST and nuclei were counterstained with DAPI for 5 min.

### Plastic Sectioning

Embryos were fixed in 4% glutaraldehyde in 25 mM sodium phosphate buffer (pH 6.8) for 4 hours at room temperature, washed twice in phosphate buffer for 30 min and dehydrated through ethanol series (20%, 40%, 60%, 80% and 99%) for 30 min. Then embryos were infiltrated and embedded with Historesin (Leica) according to manufacture. Plastic sections with thickness 5 µm were done using Leica machine. Tissue sections were stained with toluidine blue for 1 min.

### Confocal Microscopy

Confocal imaging of zebrafish embryos was performed using the Zeiss LSM510 confocal microscope. The time-lapse imaging was performed using the Olympus FluoView FV1000 confocal microscope equipped with temperature controlling chamber. Dechorionated embryos were mounted in 0.5% low-temperature melting agarose in an imaging chamber prepared from a modified Petri dish. The embryo medium was supplemented with Tricaine to inhibit movement of fish. Embryos held in the imaging chamber maintained heartbeat and circulation throughout the imaging period. Two-dimensional or three-dimensional reconstructions of image data were prepared using the standard LSM or Olympus software package and ImageJ (NIH, USA).

### Morpholino Injection

Zic6 knockdown was performed using a translation-blocking antisense morpholino oligonucleotide (MO) purchased from Gene Tool, LLC. The MO sequence was 5′-TTG GTT GAT TTG CCA AGC CCT CAG C-3′. A mismatch antisense MO with the following sequence 5′-TTG cTT GAT gTG CCt AGC tCT gAG C-3′ was used as a control. Embryos were injected with 2.1 ng Zic6 MO or 8.5 ng Zic6 mismatch MO at the 1–2 cell stage. For the rescue experiments Zic6 MO was co-injected with 25 pg of *zic6* mRNA without morpholino binding site. Results of morpholino experiments are presented in Figure S3.

### Ventricular Injections of Small Molecule Inhibitors

Injection of small molecule inhibitors in specific concentration (see Table S1) was performed at 30 hpf into the hindbrain ventricle from where it spread with the cerebrospinal fluid into the central canal [58] and their effect on the roof plate cells elongation was scored at 72 hpf under compound fluorescent microscope.

## Supporting Information

Figure S1 **Mutant analysis of RP formation.** Immunofluorescent staining of the transverse sections of the spinal cord of *smu* (A-C) and *syu* (D-F) mutants. Confocal images of the spinal cord of *smu* (M), *syu* (N) and *oep* (O) mutants at 72 hpf. Orthogonal optical sections of the confocal images of the spinal cord of *smu* (G, J), *syu* (H, K) and *oep* (I, L) mutants at different developmental stages. (P), The length of RP process in different mutants.
(TIF)

Figure S2 **Expression pattern of** ***zic6.*** (A), RT-PCR analysis of *zic6* expression. Each even line represents negative control (minus RT). MBT, mid-blastula transition. (B–E), Whole-mount *in situ* hybridization with *zic6* RNA probe at different developmental stage. c, cerebellum; de, diencephalon; h, hindbrain; m, midbrain; mhb, midbrain-hindbrain boundary; ot, optic tectum; rp, roof plate; s1, approximate position of the 1st somite; t, thalamus; te, telencephalon.
(TIF)

Figure S3 **Zic6 morpholino knock-down experiments.** (A–C), The morphants developed the curled-down body axis, the abnormal hindbrain reminiscent of that in *mib* mutants, cardiac edema. (D), Zic6 morpholino knock-down experiments (Exp 1 to 3) and rescue experiment (Exp 4). The roof plate “gap” phenotype was scored at 72 hpf in separate experiments (Exp 5 to 8, the numbers of assessed embryos are shown in brackets).
(TIF)

Table S1 **Effect of small molecule inhibitors on roof plate extension.**
(DOC)

Movie S1 **The low-resolution movie shows the spinal cord of SqET33 zebrafish (36–72 hpf) with coordinated stretching of the roof plate.**
(MOV)

Movie S2 **The high-resolution movie shows the spinal cord of SqET33-E20 zebrafish (47–65 hpf) with coordinated stretching of the roof plate.** It illustrates the barrier function of the spinal cord as shown by failed attempts of the GFP-positive neuron to cross the midline.
(MOV)
