# Supplementary figures and images for: Stretching Morphogenesis of the Roof Plate and Formation of the Central Canal
Source: PLoS One. 2013 Feb 7;8(2):e56219. doi: 10.1371/journal.pone.0056219 (PMC3567028; doi:10.1371/journal.pone.0056219)

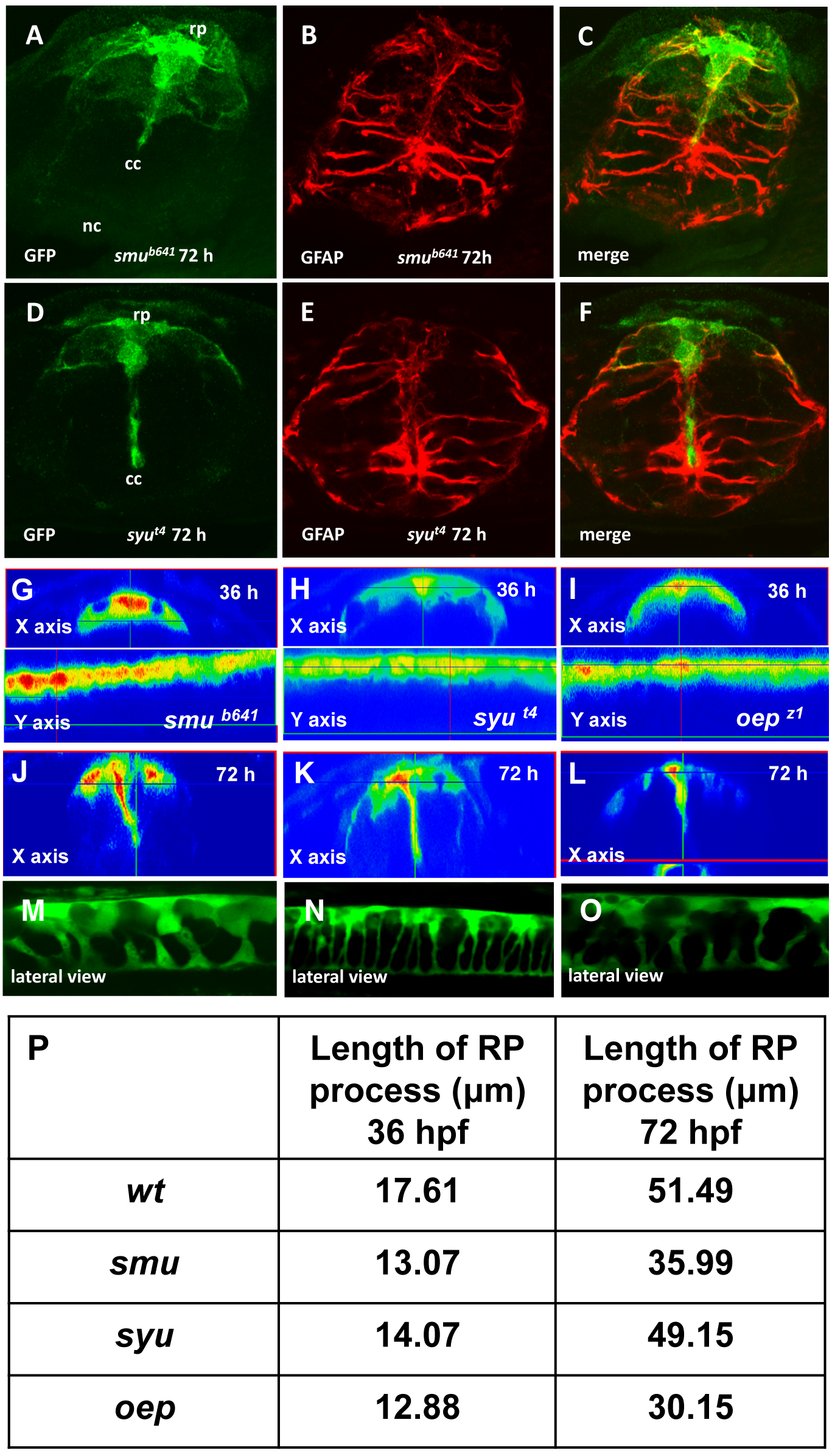

Supplement: Figure S1 — Mutant analysis of RP formation. Immunofluorescent staining of the transverse sections of the spinal cord of smu (A-C) and syu (D-F) mutants. Confocal images of the spinal cord of smu (M), syu (N) and oep (O) mutants at 72 hpf. Orthogonal optical sections of the confocal images of the spinal cord of smu (G, J), syu (H, K) and oep (I, L) mutants at different developmental stages. (P), The length of RP process in different mutants. (TIF) [file pone.0056219.s001.tif]

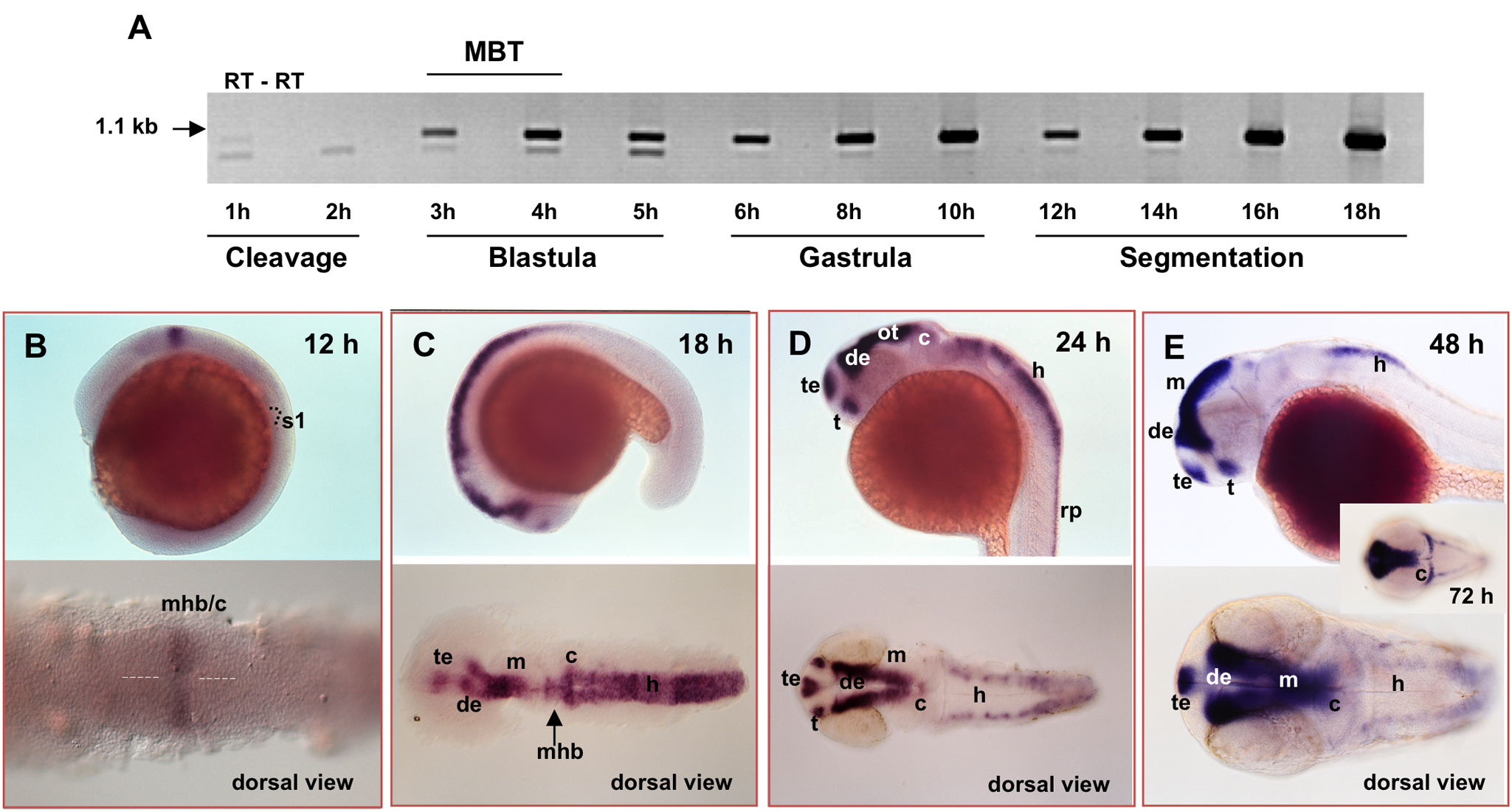

Supplement: Figure S2 — Expression pattern of zic6. (A), RT-PCR analysis of zic6 expression. Each even line represents negative control (minus RT). MBT, mid-blastula transition. (B–E), Whole-mount in situ hybridization with zic6 RNA probe at different developmental stage. c, cerebellum; de, diencephalon; h, hindbrain; m, midbrain; mhb, midbrain-hindbrain boundary; ot, optic tectum; rp, roof plate; s1, approximate position of the 1st somite; t, thalamus; te, telencephalon. (TIF) [file pone.0056219.s002.tif]

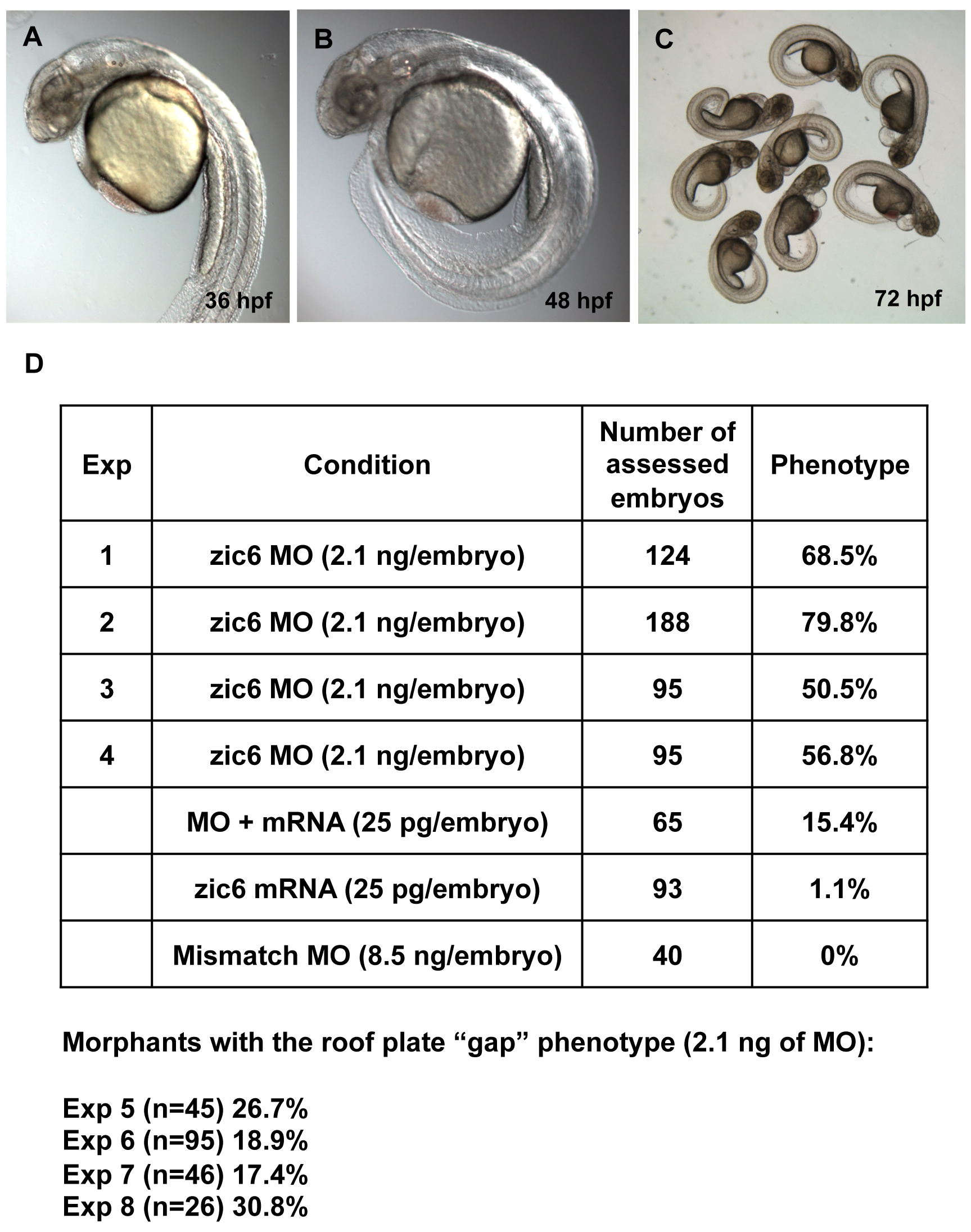

Supplement: Figure S3 — Zic6 morpholino knock-down experiments. (A–C), The morphants developed the curled-down body axis, the abnormal hindbrain reminiscent of that in mib mutants, cardiac edema. (D), Zic6 morpholino knock-down experiments (Exp 1 to 3) and rescue experiment (Exp 4). The roof plate “gap” phenotype was scored at 72 hpf in separate experiments (Exp 5 to 8, the numbers of assessed embryos are shown in brackets). (TIF) [file pone.0056219.s003.tif]
