# Supplementary material for: Stretching Morphogenesis of the Roof Plate and Formation of the Central Canal
Source: PLoS One. 2013 Feb 7;8(2):e56219. doi: 10.1371/journal.pone.0056219 (PMC3567028; doi:10.1371/journal.pone.0056219)
Supplement: Table S1 — Effect of small molecule inhibitors on roof plate extension. (DOC) [file pone.0056219.s004.doc]

**Table S1. Effect of small molecule inhibitors on roof plate extension.**

| Compound | Target | Concentration (mM) | Number of injected embryos | Phenotype |
| --- | --- | --- | --- | --- |
| Blebbistatin | Myosin-2 | 1 | 11 | no phenotype |
| Cytochalasin D | Actin | 1 | 10 | no phenotype |
| Dorsomorphin | BMP pathway | 1 | 5 | curled-up body (2/5), no "gap" in RP |
| IWR-1 | Wnt pathway | 3 | 10 | no phenotype |
| Latrunculin B | Actin | 1 | 10 | no phenotype |
| Nocodazole | Microtubules | 1 | 2 | no phenotype |
|  |  | 5 | 10 | no phenotype |
| NU-1025 | PARP | 1 | 10 | no phenotype |
|  |  | 3 | 8 | curled-up body (3/8), no "gap" in RP |
| Phalloidin | F-actin | 1 | 10 | no phenotype |
| Pyrvinium pamoate | Wnt pathway | 3 | 10 | "gap" in roof plate (1/10) |
| SU5402 | FGFR, VEGF | 1 | 10 | curled-up body and "gap" in RP (1/10) |
|  |  | 2 | 2 | no phenotype |
| Tetraethylammonium | K^+^ channel | 3 | 10 | no phenotype |
| Y27632 | Rock | 0.06 | 5 | no phenotype |
|  |  | 0.6 | 5 | curled-up body and "gap" in RP (1/5) |
|  |  | 1 | 5 | curled-up body and "gap" in RP (2/5) |
|  |  | 2 | 6 | curled-up body and "gap" in RP (3/6) |
|  |  | 6 | 5 | curled-up body and "gap" in RP (5/5) |
